# Outcome Reporting Bias in COVID-19 mRNA Vaccine Clinical Trials

**DOI:** 10.3390/medicina57030199

**Published:** 2021-02-26

**Authors:** Ronald B. Brown

**Affiliations:** School of Public Health and Health Systems, University of Waterloo, Waterloo, ON N2L3G1, Canada; r26brown@uwaterloo.ca

**Keywords:** mRNA vaccine, COVID-19 vaccine, vaccine efficacy, relative risk reduction, absolute risk reduction, number needed to vaccinate, outcome reporting bias, clinical epidemiology, critical appraisal, evidence-based medicine

## Abstract

Relative risk reduction and absolute risk reduction measures in the evaluation of clinical trial data are poorly understood by health professionals and the public. The absence of reported absolute risk reduction in COVID-19 vaccine clinical trials can lead to outcome reporting bias that affects the interpretation of vaccine efficacy. The present article uses clinical epidemiologic tools to critically appraise reports of efficacy in Pfzier/BioNTech and Moderna COVID-19 mRNA vaccine clinical trials. Based on data reported by the manufacturer for Pfzier/BioNTech vaccine BNT162b2, this critical appraisal shows: relative risk reduction, 95.1%; 95% CI, 90.0% to 97.6%; *p* = 0.016; absolute risk reduction, 0.7%; 95% CI, 0.59% to 0.83%; *p* < 0.000. For the Moderna vaccine mRNA-1273, the appraisal shows: relative risk reduction, 94.1%; 95% CI, 89.1% to 96.8%; *p* = 0.004; absolute risk reduction, 1.1%; 95% CI, 0.97% to 1.32%; *p* < 0.000. Unreported absolute risk reduction measures of 0.7% and 1.1% for the Pfzier/BioNTech and Moderna vaccines, respectively, are very much lower than the reported relative risk reduction measures. Reporting absolute risk reduction measures is essential to prevent outcome reporting bias in evaluation of COVID-19 vaccine efficacy.

## 1. Introduction

Using messenger RNA (mRNA) in vaccines to produce proteins that trigger an immune response against infectious diseases has held promise for decades, but until recently, no clinically tested mRNA vaccine has managed to advance beyond small, early-phase trials [1]. Normally, genetic code in mRNA is transcribed from DNA in the cell nucleus, and the coded message is delivered by mRNA to cell ribosomes for translation during protein biosynthesis [2]. COVID-19 mRNA vaccines directly inject cells with a synthetic genetic code to replicate the spike S protein found on the surface of the coronavirus, SARS-CoV-2 [3]. Once replicated, the spike protein is proposed to trigger an immune response that creates antibodies against the virus [4].

However, several biological obstacles continue to challenge the development of mRNA vaccines, including “mRNA’s extremely large size, charge, intrinsic instability, and high susceptibility to enzymatic degradation” [5]. To mitigate enzymatic degradation, mRNA in the vaccines is encapsulated in lipid nanoparticles [6], but it is unclear how this encapsulation affects genetic code translation in the cell ribosomes. Nevertheless, clinical results of phase III trials reported for COVID-19 vaccines manufactured by Pfizer/BioNTech (New York City, NY, USA/Mainz, Germany) [7] and Moderna (Cambridge, MA, USA) [8] have far surpassed predicted performance, with vaccine efficacy rates of approximately 95%. Curiously, “why these vaccines seem so effective while previous attempts against other pathogens haven’t appeared as promising remains an open question” [1].

As noted in *BMJ Opinion*, 26 November 2020 [9],

“There may be much more complexity to the ‘95% effective’ announcement than meets the eye—or perhaps not. Only full transparency and rigorous scrutiny of the data will allow for informed decision making. The data must be made public.”

As was also noted in the *BMJ Opinion*, Pfizer/BioNTech and Moderna reported the relative risk reduction of their vaccines, but the manufacturers did not report a corresponding absolute risk reduction, which “appears to be less than 1%” [9]. Absolute risk reduction (ARR) and relative risk reduction (RRR) are measures of treatment efficacy reported in randomized clinical trials. Because the ARR and RRR can be dramatically different in the same trial, it is necessary to include both measures when reporting efficacy outcomes to avoid outcome reporting bias. In the present article, a critical appraisal of publicly available clinical trial data verifies that absolute risk reduction percentages for Pfizer/BioNTech vaccine BNT162b2 [7] and Moderna vaccine mRNA-1273 [8] are, respectively, 0.7%; 95% CI, 0.59% to 0.83%; *p* = 0.000, and 1.1%; 95% CI, 0.97% to 1.32%; *p* = 0.000. The same publicly available data, without absolute risk reduction measures, were reviewed and approved by the roster of members serving on the U.S. Food and Drug Administration’s (FDA’s) Vaccines and Related Biological Products Advisory Committee (VRBPAC) for emergency use authorization (EUA) of the mRNA vaccines [10]. Ironically, the omission of absolute risk reduction measures in data reviewed by the VRBPAC overlooks FDA guidelines for communicating evidence-based risks and benefits to the public [11]. The FDA’s advice for information providers includes:

“Provide absolute risks, not just relative risks. Patients are unduly influenced when risk information is presented using a relative risk approach; this can result in suboptimal decisions. Thus, an absolute risk format should be used.”

The *New England Journal of Medicine* also published clinical trial data on safety and efficacy for the BNT162b2 vaccine [12] and the mRNA-1273 vaccine [13], but with no mention of absolute risk reduction measures.

The present article uses epidemiologic tools to critically appraise absolute and relative risk reduction measures for vaccine efficacy in phase III clinical trials of the COVID-19 mRNA vaccines. Microsoft Excel was used to analyze data and chart risk reduction outcomes. The article further clarifies how selective reporting of vaccine efficacy measures can cause a type of outcome reporting bias that misrepresents health information disseminated to the public.

## 2. Critical Appraisal of Vaccine Efficacy

The application of epidemiologic and biometric methods to clinical diagnosis and treatment is known as clinical epidemiology [14]. Clinical epidemiologic tools can be applied in evidence-based medicine (EBM) to critically appraise research evidence for validity, size of effect, and usefulness in clinical practice [15]. Clinical treatment effects in groups of participants are measured by comparing probabilities of an event, known as event rates [16].

**Figure 1:**
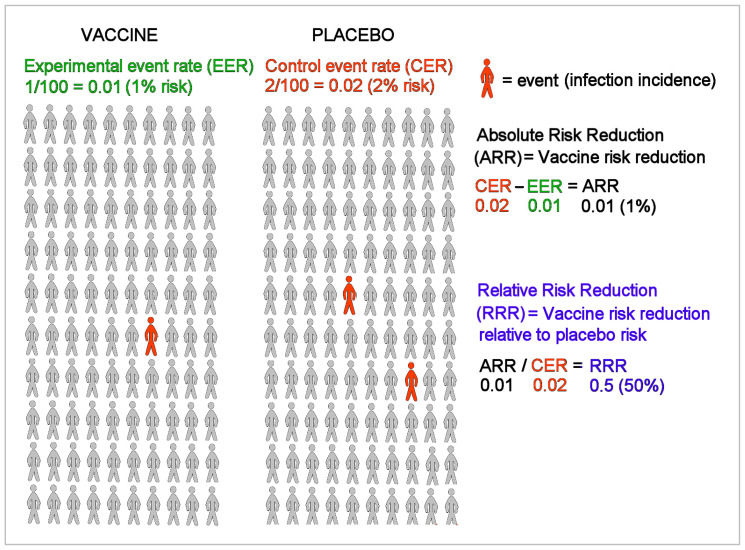
Example of a vaccine clinical trial for an infectious disease.

Figure 1 shows an example of a vaccine clinical trial for an infectious disease. The vaccine and placebo groups in Figure 1 each have 100 randomly assigned individuals with no history of infection, and an event is defined as the incidence of infection among all individuals during the course of the trial. The percentage of events in the vaccine group is the experimental event rate (EER) or the risk of infection in the vaccine group (1/100 = 1%), and the percentage of events in the placebo group is the control event rate (CER) or the risk of infection in the placebo group (2/100 = 2%). Absolute risk reduction (ARR) is the disease risk difference between the placebo and vaccine groups, i.e., the CER minus the EER (2% − 1% = 1%). The ARR is also known as the vaccine disease preventable incidence (VDPI) [17]. Relative risk reduction (RRR) or vaccine efficacy (VE) is the reduced risk from vaccination, the ARR or VDPI, relative to or divided by the risk in unvaccinated individuals, the CER (1%/2% = 50%) [18].

## 3. 2 × 2 Contingency Tables and Epidemiologic Equations

The following 2 × 2 contingency tables for SARS-CoV-2 infection are based on reported clinical trial data for the Pfzier/BioNTech BNT162b2 vaccine [12] and the Moderna mRNA-1273 vaccine [13]. The table rows, shown in Table 1, list the vaccine and placebo groups and the table columns list the participants’ outcomes of either SARS-CoV-2 infection or no infection. Table 2 and Table 3 list the clinical trial data for the Pfzier/BioNTech and Moderna vaccines, respectively. As shown in Table 1, the total number of participants in a group, known as *n*, is represented by a + b for the vaccine group and c + d for the placebo group.

The following epidemiologic equations use data from the 2 × 2 contingency tables (Table 1, Table 2 and Table 3) to calculate relative and absolute measures of COVID-19 mRNA vaccine efficacy.

Risk ratio (RR):

(1) $$\;\mathrm{RR}\;=\;\frac{a/\left(a+b\right)}{c/\left(c+d\right)}$$

The risk ratio, also known as the relative risk, in a randomized controlled trial is the ratio calculated by dividing the experimental event rate (EER), *a*/(*a* + *b*), by the control event rate (CER), *c*/*(c* + *d*) [19]. Dividing the EER by the CER equals 1 if the rates do not differ, in which case the RR has the null value 1. RRs below 1 indicate a protective effect and a decreased risk (EER < CER), and RRs above 1 indicate an increased risk (EER > CER).

Risk ratio 95% confidence interval (CI):

(2)  CI = e^(Ln(RR) ± 1.96∗SE) where SE = b/a(a+b)+d/c(c+d) or 1a−1(a+b)+1c−1(c+d)  

The risk ratio 95% confidence interval predicts the range of probable risk ratios if the experiment or trial was repeated 95 out of 100 times. The narrower the range between the upper and lower CI values, the more precise the CI. If the range includes the RR null value, 1, the risk ratio is considered statistically insignificant. The equation calculates the standard error (SE) [20,21], and the natural logarithm (Ln) is used, along with the antilog expressed as an exponent of the base e, to normally distribute the data when calculating the 95% probability.

Absolute risk reduction (ARR):

(3) $$\mathrm{ARR}\;(\%)\;=\;\frac{c}{\left(c+d\right)}-\;\frac{a}{\left(a+b\right)}$$

The absolute risk reduction is a percentage equal to the arithmetic difference when subtracting the EER from the CER [19]. The difference equals zero if the rates do not differ, in which case the ARR has the null value zero. The difference is negative if the EER is higher than the CER.

Absolute risk reduction 95% confidence interval (CI upper, lower):

(4) $$\mathrm{ARR}\;\mathrm{CI}\;=\;\mathrm{ARR}\;\pm \;1.96*\mathrm{SE},\;\mathrm{where}\;\mathrm{SE}\;=\;\sqrt{\frac{\mathrm{EER}*\left(1\;-\;\mathrm{EER}\right)}{\left(a+b\right)}\;+\;\frac{\mathrm{CER}*\left(1\;-\;\mathrm{CER}\right)}{\left(c+d\right)}}$$

The standard error in the absolute risk reduction 95% confidence interval measures the square root of the sum of the group variances [22]. If the ARR CI includes the null value zero, the ARR is not statistically significant.

Number needed to vaccinate (NNV):

(5) $$\mathrm{NNV}\;=\;\frac{1}{\mathrm{ARR}}$$

The NNV, or the number needed to vaccinate to prevent one infection, is the reciprocal of the ARR [17]. Note that the numerator is multiplied by 100 when the ARR is expressed with a percentage sign. The NNV is also usually rounded up to the next individual.

NNV 95% confidence interval (CI):

(6) $$\mathrm{NNV}\;\mathrm{CI}\;=\;\frac{1}{\mathrm{ARR}\;\mathrm{CI}}\;$$

The CI of the NNV is calculated by dividing 1 by the ARR CI [22], again multiplying by 100 in the numerator when the ARR is expressed with a percentage sign.

Relative risk reduction (RRR) or vaccine efficacy (VE):

(7) RRR, VE (%) = 1 − RR

The relative risk reduction is the same as vaccine efficacy (VE) [17]. The RRR is calculated by subtracting the RR from the null value 1, or by dividing the ARR by the CER [22].

RRR, VE 95% confidence interval (CI):

(8) RRR, VE CI = 1 − RR CI

The CI for the relative risk reduction is calculated by subtracting the RR CI from the null value 1.

**Figure 2:**
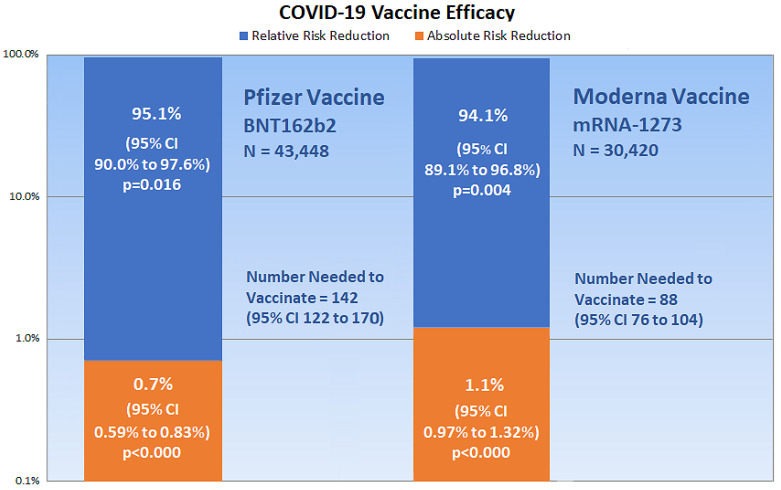
The chart shows critical appraisal results of mRNA COVID-19 vaccine efficacy.

Pvalues, which measure the probability that a trial result occurred by chance, can be calculated from the confidence interval for the difference between two proportions, as in the ARR, and from the confidence interval for a ratio, as in the RRR [23]. Online calculators are also available that compare group proportions [24] and calculate epidemiological equations [25], which are useful for measuring vaccine efficacy. Figure 2 shows a chart of the present critical appraisal of mRNA COVID-19 vaccine efficacy. Note that the vertical axis of the chart is a logarithmic scale, base 10.

Clinical epidemiologic tools can be used to critically appraise the efficacy of new COVID-19 vaccines having biological mechanisms that differ from the mRNA vaccines, such as AstraZeneca-Oxford’s ChAdOx1 adenoviral vector vaccine [26] and Johnson & Johnson’s Janssen Biotech Ad26.COV2.S vaccine [27]. (As this article goes to press, the FDA VRBPAC is scheduled to review the Janssen Biotech vaccine for EUA.) As well, reported efficacy for randomized clinical trials involving any treatment, intervention, disease, disorder, or illness can be critically appraised using clinical epidemiologic tools. In a similar manner, observational studies that report vaccine and other treatment effectiveness in reducing disease incidence within a population can also be critically appraised using clinical epidemiologic tools.

## 4. Discussion

Medical and public health experts continue to stress the need to include measurements of absolute risk reduction and number needed to treat when reporting results of clinical interventions [28]. Currently, differences between relative effect measures and absolute effect measures in studies are “poorly understood by health professionals, and even more poorly understood by patients.” [29] In addition,

“…critical appraisal knowledge and skills are limited among physicians,” and “use of relative effect measures was associated with greater perceptions of medication effectiveness and intent to prescribe, compared with the use of absolute effect measures.”

[29]

Reporting relative measures may be sufficient to summarize evidence of a study for comparisons with other studies, but absolute measures are also necessary for applying study findings to specific clinical or public health circumstances [22]. Omitting absolute risk reduction findings in public health and clinical reports of vaccine efficacy is an example of outcome reporting bias, which ignores unfavorable outcomes and misleads the public’s impression and scientific understanding of a treatment’s efficacy and benefits [30]. Furthermore, the ethical and legal obligation of informed consent requires that patients are educated about the risks and benefits of a healthcare procedure or intervention [31].

Similar to the critical appraisal in the present article, critical appraisals of reported vaccine efficacy in other studies reveals clinically significant insights. For example, a 2018 review of 52 randomized trials for influenza vaccines that studied over 80,000 healthy adults reported an overall influenza vaccine EER of 0.9% and a 2.3% CER, which calculates to a RRR of 60.8% [32]. This vaccine efficacy is consistent with a 40% to 60% reduction in influenza reported by the Centers for Disease Control and Prevention (CDC) [33]. However, critically appraising data from the 2018 review shows an overall ARR of only 1.4%, which reveals vital clinical information that is missing in the CDC report. A 1.4% ARR works out to a NNV of approximately 72 people, meaning that 72 individuals need to be vaccinated to reduce one case of influenza. By comparison, Figure 2 of the present article shows that the NNV for the Pfzier-BioNTech and Moderna vaccines are 142 (95% CI 122 to 170) and 88 (95% CI 76 to 104), respectively.

The mRNA vaccine manufacturers reported that infections in most subgroups in phase III clinical trials were similar for both vaccines after two doses. Vaccine clinical trial case definitions for SARS-CoV-2 infection included COVID-19 clinical symptoms; thus the trials were not designed to provide evidence of vaccine efficacy for protection against asymptomatic infections. In addition to outcome reporting bias, information bias may have also affected COVID-19 vaccine trial outcomes due to misclassification of SARS-CoV-2 infections as mild adverse effects of the vaccines. For example, several COVID-19 clinical symptoms are similar to the vaccines’ adverse effects such as fever, pain, and fatigue, which could potentially lead to missed diagnoses of viral infections.

A limitation of this article is that it only critically appraised mRNA vaccine efficacy in healthy individuals who were randomized to two groups under strictly controlled conditions. The critical appraisal did not include vaccine safety and effectiveness outcomes within a general population that includes unhealthy people and that lacks control over confounding factors. For example, healthy vaccinee bias occurs when people who are in better health are more likely to follow vaccination recommendations in order to protect their health [34].

## 5. Conclusions

A critical appraisal of phase III clinical trial data for the Pfizer/BioNTech vaccine BNT162b2 and Moderna vaccine mRNA-1273 shows that absolute risk reduction measures are very much lower than the reported relative risk reduction measures. Yet, the manufacturers failed to report absolute risk reduction measures in publicly released documents. As well, the U.S FDA Advisory Committee (VRBPAC) did not follow FDA published guidelines for communicating risks and benefits to the public, and the committee failed to report absolute risk reduction measures in authorizing the BNT162b2 and mRNA-1273 vaccines for emergency use. Such examples of outcome reporting bias mislead and distort the public’s interpretation of COVID-19 mRNA vaccine efficacy and violate the ethical and legal obligations of informed consent.

## Figures and Tables

**Table 1:** 2 × 2 contingency table for SARS-CoV-2 infection in vaccine clinical trials.

	Infection	No Infection	
Vaccine	a	b	a + b
Placebo	c	d	c + d

**Table 2:** 2 × 2 contingency table for SARS-CoV-2 infection in Pfzier/BioNTech vaccine clinical trial.

	Infection	No Infection	
BNT162b2	8	21,712	21,720
Placebo	162	21,564	21,726

**Table 3:** 2 × 2 contingency table for SARS-CoV-2 infection in Moderna vaccine clinical trial.

	Infection	No Infection	
mRNA-1273	11	15,199	15,210
Placebo	185	15,025	15,210

## Data Availability

Data for Pfzier/BioNTech BNT162b2: https://doi.org/10.1056/nejmoa2034577; data for Moderna mRNA-1273: https://doi.org/10.1056/NEJMoa2035389 (accessed on 10 January 2021).
